# Applications of Extreme Value Theory in Corrosion Engineering

**DOI:** 10.6028/jres.099.028

**Published:** 1994

**Authors:** Philip A. Scarf, Patrick J. Laycock

**Affiliations:** University of Salford, Salford M5 4WT, U.K.; University of Manchester Institute of Science and Technology, Manchester M60 1QD, U.K.

**Keywords:** corrosion, exccedanccs, extreme values, extreme value distributions, generalized Parcto distribution

## Abstract

In the context of corrosion engineering it is often natural to be concerned with extreme events. This is because, firstly, it is these extreme events that often lead to failure and, secondly, it may only be possible to measure the extremes, with much of the underlying measurements by their very nature unobservable Statistical methods relating to extreme value theory can be used to model and predict the statistical behaviour of extremes such as the largest pit, thinnest wall, maximum penetration or similar assessment of a corrosion phenomenon. These techniques can be applied to the single largest value, or to a given number of the largest values, measured over individual areas or coupons; or to all values exceeding a given threshold. The data can be modeled to account for dependence on environmental conditions, surface area examined, and the duration of exposure or of experimentation. The application of a selection of these techniques is demonstrated on data from industry and from laboratory experiments.

## 1. Introduction

Extremes are typically defined in two ways. Either by selecting a suitable threshold and then recording every observation above that threshold; or by sorting the data, according to some *a priori* sampling scheme, so as to select the one, two, or three, etc., largest value(s). The nature by which the extremes are defined and hence measured is then indicative of the techniques appropriate for modeling and prediction. Most of the statistical methods relating to extreme values are based, in the first instance, on the assumption of an underlying large sample of possible measurements, all nominally arising from a single population of such possible measurements. For extreme value theory to be used, it is then only necessary for the actual extremes to be measured. The other possible measurements can be ignored and may even be unobservable with the equipment used to measure the extremes. The nature of the extreme may be that of a maximum value or a minimum value. In this paper we will assume that maximum values are of interest. In applications concerned with minima, negating the variable of interest will transform the problem into one concerned with maxima.

The generalized Pareto distribution (GPD) is the standard family of statistical distributions to be used as a basis for modeling data which arise as exceedances over some threshold. Applications of this approach for the first of the above extreme value definitions is examined in the following section. Methods to ensure the validity of the standard statistical assumptions while accumulating such data are discussed. The generalized extreme value (GEV) distribution can be shown to be the natural one to use for single extremes. Data can arise as the largest value from each of a set of coupons (individual specimens), or from partitioning an area into equal smaller areas and selecting one maximum from each smaller area. The application of methods considering such single extremes is also considered. The joint generalized extreme value distribution (JGEV) is the appropriate distribution family to use when the *r* (say) largest values are extracted, instead of just the single largest value. This provides a useful extension to the classical theory in such a way as to match up with the common practice of measuring the few largest pits at any one location undergoing pitting. Using the *r* extreme order statistics in this way can increase the precision of the estimates in the model and hence improve predictions.

Dependence on time and area can be incorporated for prediction and extrapolation purposes when applying these distributions, and methods for modeling the dependence on environmental conditions, say, through covariates are indicated.

## 2. Exceedances Above a Threshold

These are data collected on the basis of all values exceeding a specified threshold, taken sufficiently “high” to imply that certain limiting statistical results will hold. The data in Table 1, on pit depths in two stainless steel roofs, were collected with just such a threshold, namely 6 μm, in operation. This threshold qualifies as “high” on the basis that a much lower one, such as 0.06 μm for example, would have produced a very much larger sample of nascent pits. This is consistent with theories of pitting in steel and other metals. See further argument supporting this approach in Ref. [1]. This type of data censoring can arise through built in limits on measurement capabilities or else through deliberate censoring of a given data set, typically a dense time series, so as to isolate the important events. When such data are extracted from a regular grid of values rather than through the engineer visually identifying isolated corrosion phenomena and taking one measurement on each, it may be necessary to edit the values so as to extract only local cluster maxima rather than using all nearby points. This is needed to “decouple” the recorded values and so validate the usual assumption of statistical independence or exchangeability. A careful combination of grid size (to match the scale of the phenomena being studied) and threshold (to select for significant phenomena) may be all that is necessary.

With this form of data set, both the number, *n*, of observations and their observed values {*y_i_*} are necessarily random variables. It can be shown, see for example Ref. [2], that, for sufficiently high thresholds, and for a wide variety of initial distributions, this number, *n*, of the exceedances, has asymptotically a Poisson distribution (with parameter λ, say) and their sizes, *y*, have a generalized Pareto distribution:

$$G\left(y\right)=1-{\left(1+\xi y/\sigma \right)}^{-1/\xi },$$ (1)

valid for 1 + *ξy/σ* > 0, with *σ* > 0 and − ∞ < *ξ* < ∞. In particular, if these distributional results hold exactly for some particular threshold, *u* say, then the maximum of this set of values has a generalized extreme value distribution (see next section) exactly, and this will be true for all higher thresholds. A check that the distribution, Eq. (1), holds can be made by graphing the mean excess plot, in which the mean exceedances in the data are plotted against increasing threshold values. This plot should follow a straight line with slope *ξ*/(l − *ξ*) and intercept *σ*/(l − *ξ*); with a horizontal plot corresponding to *ξ* = 0 and a simple exponential distribution for the tail. For extrapolation over larger areas, for extremes derived from random sampling over a large structure, often the quantity of interest is the *N*th return level

$$q_{N}=u-\frac{\sigma }{\xi }\left[1-{\left(\lambda N\right)}^{\xi }\right],$$

where *N* is either the number of “coupon multiples” as a measure of structure size, or else the number of time intervals into the future. The *N*th return level is interpreted as that level which would be exceeded on average once every *N* units of area (or time).

The data in Fig. 1(a) are 1024 values of “current noise” collected during a study of the electrochemical nature of pitting. This series was “declustered” using a moving window of width 40 to give the isolated maxima in Fig. 1(b). A mean excess plot for the isolated maxima of the current noise data is given in Fig. 1(c). Consideration of this plot suggests that either a large threshold is required or that the exceedances arise from a mixture of the tails of underlying distributions. For an electrochemical interpretation of this latter phenomenon, it can be noted that large narrow current spikes have been described as being typical of intermittent pitting corrosion, while steady broader based but less variable current noise has been associated with general corrosion, see for example Ref. [3]. Intermediate conditions can be associated with persistent pitting, widely recognized as the most threatening scenario for metal structures.

The main difficulty which can arise with the threshold method is the choice of an appropriate threshold, especially when there is no *a priori* reason for choosing one particular threshold over another. In an experiment to consider the prediction of extreme corrosion rates for carbon steel in a simulated basalt groundwater [4], a number of 200 mm × 200 mm coupons were exposed for varying lengths of time. These coupons, having been first cleaned to remove all corrosion products, were profiled with spot heights taken at the nodes of a 1 mm lattice. This then gave, after making an adjustment for the original coupon surface, a 196 × 196 array of corrosion measurements. False-color histogram-equalization techniques, displayed on computer monitors, were used to validate and inspect the digitized spot heights from these coupons. A mean excess plot for a typical coupon exposed for 26 weeks is shown in Fig. 2(a). Note that this plot was drawn for both the raw exceedances and also for declustered exceedances. The process of declustering essentially amounted to identifying all those “pits” or clusters exceeding a particular threshold and calculating the maximum exceedance for each “pit.” The mean excess plot indicates that a range of possible thresholds (300 μm–550 μm) would be appropriate for model fitting. Table 2 gives the results for such model fitting using maximum likelihood for a range of values of threshold. Here λ is the mean exceedance rate per m^2^, *σ*, and *ξ* are the parameter estimates for the GPD, and *q*_25_ and *q*_250_ are those levels that would be exceeded once on average every m^2^ and every 10 m^3^ respectively. Standard errors are given in brackets. If the *q*_25_ is considered, we see that its estimated value decreases as the threshold increases, its value being highly sensitive to the value of *ξ*. For higher thresholds the large negative value of *ξ* is indicative of a tail distribution which is shorter than exponential so implying lower return values. For lower thresholds the tail appears to be exponential implying relatively higher return values. This effect can be seen further in an exponential probability plot of the exceedances above 300 μm, Fig. 2(b). As the threshold increases more weight is given to the extreme observations, which are themselves smaller than would be expected for an exponential tail. The lack of an objective method for determining the correct threshold therefore leads to difficulties in prediction.

## 3. Extreme Value Distributions

Data suitable for this type of analysis can arise as the largest value from each of a set of coupons, or from dividing an area into equal smaller areas and selecting one maximum from each smaller area, provided the scale of division and corrosion patterns are compatible in the sense described above for the generalized Pareto distribution. For a sample of independent identically distributed random variables, *x*_1_,…*x_n_*, the distribution of *x*_max_, the data maximum, depends on *n*. Suppose however that there exist location and scale factors, *a_n_* and *b_n_* say, so that the rescaled variate, *y = a_n_ + b_n_x*_(_*_n_*), has a distribution which is independent of *n*. This is the so-called “stability postulate,” and leads immediately to the following functional equation (to be solved for *F*)*: F*(*x*)*^n^ = F*(*a_n_ + b_n_x*). The solution to this equation is the generalized extreme value (GEV) distribution, which can be written in the following 3-parameter form:

$$\begin{array}{c} F(x)=\exp\{-{\left[1+\xi (x-\mu )/\psi \right]}^{-1/\xi }\},\\ \xi x>\xi \mu -\psi =\xi \theta ,\psi >0. \end{array}$$ (2)

See for example Ref, [5]. Note also that if the assumption of independence is relaxed, under general conditions the distribution, Eq. (2), is still the appropriate one for maxima. It turns out that almost all standard distributions satisfy the stability postulate asymptotically, although it is only exactly true for the GEV distribution itself. This is exactly analogous to the Central Limit Theorem for averages, which is satisfied asymptotically by almost all standard distributions, but only holds exactly for an initial Normal distribution. As with averages, which are assumed Normal, by the Central Limit Theorem, and then fitted accordingly, so with maxima, it is reasonable to assume a GEV distribution and fit accordingly. Since the dependence of the stability coefficients, *a_n_*, *b_n_*, on *n* is typically logarithmic, or slower, we can extract maxima from samples which are roughly the same size. In engineering practice this is often almost unverifiable, but nevertheless a plausible assumption, since the bulk of the data, “too small to be seen,” may be uncounted, let alone observed. The physical size of components and common conditions may be the only justification.

For extrapolation over larger areas (for extremes derived from random sampling over a large structure) or over longer time periods (for extremes derived from sampling at regular intervals of lime), the *N*th return level can be defined by solving *F*(*x*) = 1 − 1*/N*. Again *N* is interpreted as in the previous section. Alternatively, after fitting the distribution to the given data, the implied distribution of extreme values from future samples over larger areas and longer lengths of time (with equal base populations) can be deduced and properties such as the mean extreme, etc., inferred from this more fundamental approach. For a full discussion see Ref. [1], However, the return period method is particularly easy to implement for type I extreme value probability plots. For examples of these plots applied to pit depths in steels exposed to marine environments see Refs. [6,7]. The parameters can also be regressed on covariates as appropriate, to allow for dependence on measured environment variables and/or time, see for example Ref. [8]. A more subtle approach for modeling covariates would use an extreme value regression model of the sort considered in the context of the Weibull distribution [9].

**Fig. 3:**
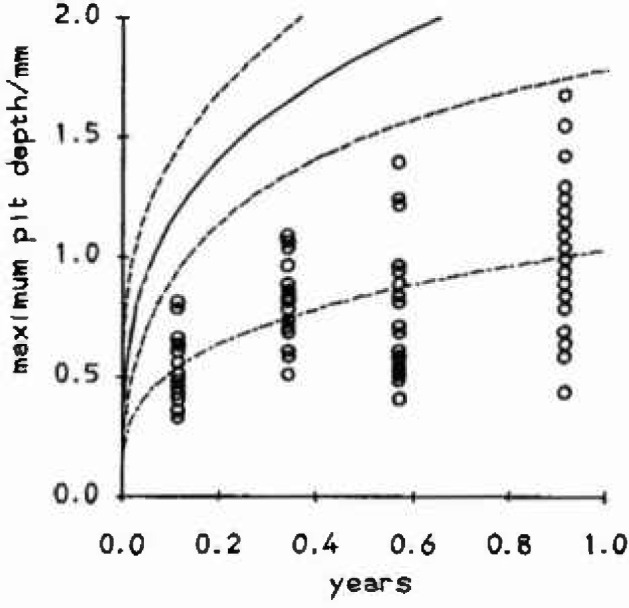
Maximum pit depths against lime for Carbon steel in alkaline conditions along with fitted mean function (−·−), upper bound (—) and confidence curves for the upper bound (---).

In Ref. [10] each of five circular coupons were exposed to a corrosive medium for each of four different exposure times: 1000 h, 3000 h, 5000 h, and 8000 h. The maximum pit depth was measured in each of six equal sectors on each specimen. Nominally this gave 120 pit depths in all, however, for many coupons, pits overlapped into a number of sectors and so the number of independent maxima was significantly reduced. Figure 3 shows a plot of maximum pit depth against exposure time for resulting data. The plotted mean function and upper bound are based on the fitting of a 4-parameter time dependent GEV distribution for which *μ = μt^β^, ψ_t_ = ψt^β^* and *ξ* is constant. This model gives

$$\begin{array}{cc} \mu_{t}=0.912\left(\pm 0.063\right)t^{\beta } & \psi_{t}=0.293\left(\pm 0.037\right)t^{\beta }\\ \beta =0.298\left(\pm 0.051\right) & \xi =-0.216\left(\pm 0.121\right) \end{array}$$

The corresponding mean function is $\eta_{t}\left[\theta +\frac{\psi }{\xi }\Gamma \left(1-\xi \right)\right]t^{\beta }=\eta t^{\beta }$, which agrees with the common assumption made in the corrosion literature of a power law growth of the mean maximum pit depth with time [8,11,12]. The implied upper bound is then *θ_t_* = *θt^β^* = (*μ* −ψ/*ξ*)*t^β^*. Such means and bounds can be extrapolated out to larger areas of exposed metal and to longer time periods using the methods described in Ref, [1], Standard errors on the upper bound were calculated by reparameterizing the problem and constructing a profile likelihood for *θ_t_* as in Ref. [2]. The negative value for the shape parameter *ξ* has been observed by the authors of this paper consistently for corrosion phenomena of many types and in many environments. This has important consequences for extrapolation since, in corrosion engineering return levels are often very large (e.g., it may only be possible to inspect a small number of one meter sections of a buried pipeline which may be hundreds of kilometers in length), and so for the range of values of *ξ* encountered by the authors, the maximum will be very close to the upper bound or end point of the distribution. This should be contrasted with the commonly used *ξ* = 0, type I extreme value distribution, [6–8, 11] for which there is no upper bound.

## 4. Extreme Order Statistics

There is a corresponding asymptotic result concerning the joint distribution of the r largest values, *x_max_ = x*_(1)_*≥ …≥x*_(_*_i_*_)_, from a sample of independent identically distributed random variables. Data will in general then consist of *m* sets of such largest values. The joint generalized extreme value distribution (JGEV) has density

$$\begin{array}{c} f\left(x_{1},x_{2},\ldots x_{r}\right)=\psi^{-r}\exp\{-[1+\frac{\xi }{\psi }\left(x_{r}-\mu \right){]}^{-1/\xi }\\ -(\frac{1}{\xi }+1)\sum_{j=1}^{r}\log[1+\frac{\xi }{\psi }(x_{j}-\mu )]\}, \end{array}$$ (3)

valid for *ξx_j_* > *ξμ − ψ = ξ0, ψ* > 0(*j* − 1,..,*r*). See for example Ref. [13]. This is the appropriate distribution to use when the *r* (say) largest values are extracted from coupons or sampled areas, instead of just the single largest value. This provides a useful extension to the classical theory in such a way as to match up with the common practice of measuring the few largest pits at any one location undergoing pitting. Using all this information rather than just the single largest extreme enables smaller confidence bands to be drawn around predicted values. However care is needed to ensure that *r* is not taken so large as to invalidate the choice of the asymptotic distribution, Eq. (3).

When *ξ* = 0, this model reduces to the Gumbel form of the JGEV with density

$$\begin{array}{c} f\left(x_{1},x_{2},\ldots x_{r}\right)=\psi^{-r}\exp\{-\exp[-\frac{1}{\psi }\left(x_{r}-\mu \right)]\\ -\sum_{j=1}^{r}\frac{1}{\psi }(x_{j}-\mu )\}. \end{array}$$ (4)

**Fig. 4:**
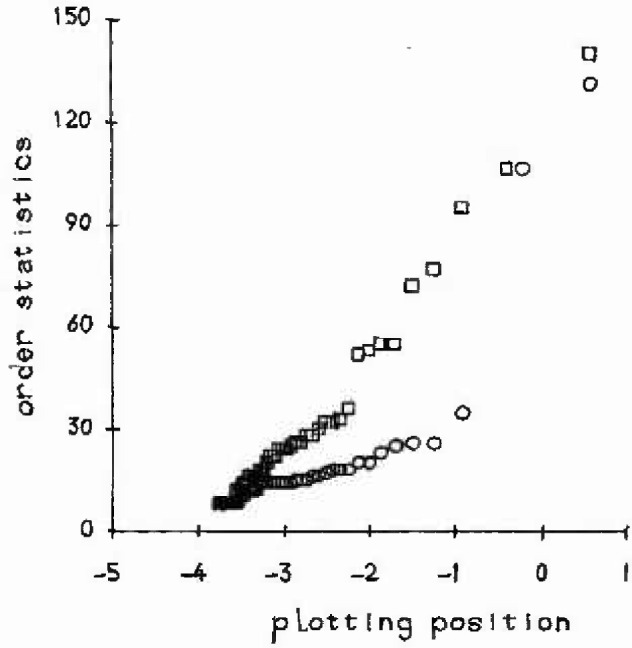
Joint Gumbel plot for the college roof data: ○–roof 1; □–roof 2.

A useful diagnostic here is the joint Gumbel plot. When *x*_(1)_*≥ …≥x*_(_*_i_*_)_ have density, Eq. (4) *E*(*x*_(_*_i_*_)_) *= μ − ψψ*(*i*) (all 1 *≤ i ≤ r*) [14], where *ϕ*(*·*) is the digamma function. Thus a plot of the order statistics *x*_(_*_i_*_)_ against − *ϕ*(*i*) will give a straight line with slope *ψ* and intercept *μ* if the Gumbel form of the JGEV distribution is appropriate. Such a plot is shown in Fig. 4 for each of the pitted college roofs data in Table 1. This plot indicates that these extremes arise from perhaps a mixture of two tail distributions. However it was assumed that *ξ* = 0 for both roofs and that for roof 1, the two largest values were to be outliers from the model, Eq. (4). These two values were removed for the purpose of analysis, and the slopes and intercepts resulting used as starting values for determining the maximum likelihood estimates of the parameters in Eq. (4). The fitted values, with their standard errors, were

$$\begin{array}{cc} \mu =54.2\left(\pm 7.9\right) & \psi =12.5\left(\pm 2.1\right),\text{roof}\;1,\\ \mu =103.2\left(\pm 15.8\right) & \psi =26.0\left(\pm 4.2\right),\text{roof}\;2. \end{array}$$

These values are then available for the implied Gumbel distribution of the maximum value, which has mean *μ* + 0.5772*ψ*. This gives 61.4 μm for roof 1 and 118.2 μm for roof 2. Extrapolation could now proceed according to the method described in the previous section, noting however that the mean of the maximum for roof 1 is considerably out of line with the observed maximum of 131 μm.

Reference [15] reports on an experiment where 15 low alloy steel specimens were suspended in a deionized warm water bath under free corrosion conditions. Specimens were removed at varying intervals up to 71 days, then after cleaning, pit depths and diameters were measured optically. A 4-parameter JGEV distribution incorporating a power law dependence on time [16] was fitted to these pit-depths, utilizing the two largest pits from each side of the specimens giving parameter values:

$$\begin{array}{cc} \mu =7.041\left(\pm 0.710\right)t^{\beta } & \psi =0.467\left(\pm 0.066\right)t^{\beta },\\ \beta =0.609\left(\pm 0.016\right) & \xi =-0.513\left(\pm 0.126\right). \end{array}$$

**Fig. 5:**
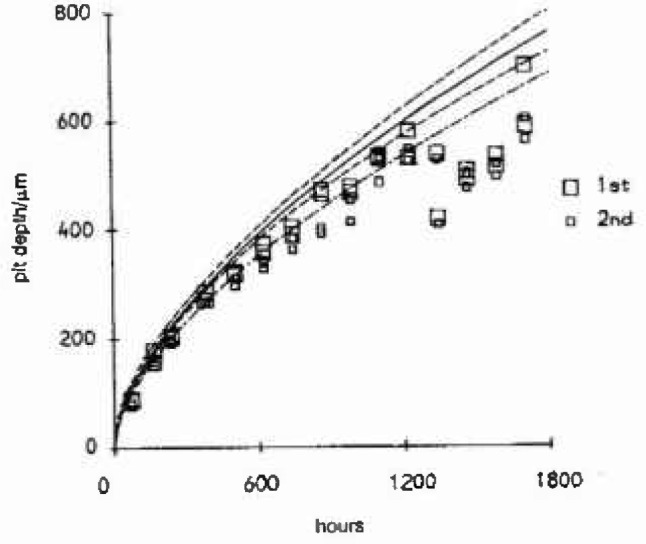
First and second largest pit depths against time for low alloy steel in deionized warm water, along with fitted mean function (−·−), upper bound (—) and confidence curves for the upper bound (---).

These are the maximum likelihood estimates for their data, for which they were only, at that time, able to report initial probability weighted moment and regression estimates. Figure 5 shows a plot of this data along with the fitted mean function and upper bound, and confidence curves for the upper bound calculated using the profile likelihood method discussed in the previous section.

## 5. Discussion

A number of statistical techniques relating to extreme value theory have been described and demonstrated on selected sets of corrosion data. Noting that much corrosion data are inherently of an extreme nature, purely statistical considerations along the lines described in this paper may be the only means of determining numerical values for prediction of the maximum pit depth in an area *A* at time *t*, for example, along with some estimate of precision or possible error. There is much evidence in the literature that *ξ* < 0 for the GEV distribution in the context of extremes of corrosion phenomena. Return levels are often very large and so, for the range of values of *ξ* encountered, predicted maxima will often be very close to the implied upper bound or end point of the distribution.

It should be noted however, that with all the methods described here, there are pitfalls. When modeling exceedances, for example, it is difficult to choose the threshold objectively, and different thresholds can lead to different predictions. Similar problems exist in the use of the *r* largest order statistics and also the maximum itself. How many largest order statistics should be used? When recording single maxima, how large should the sampled area be? While some theoretical results are available to answer such questions (e.g., Ref. [17]) these are not very helpful in a practical context.

## Figures and Tables

**Fig. 1(a):**
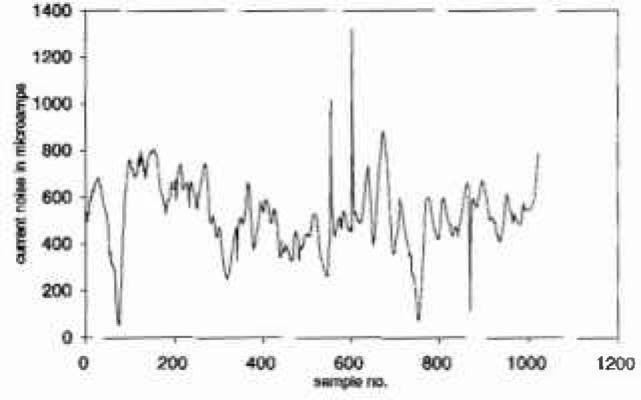
Current noise measurement (sample size = 1024).

**Fig. 1(b):**
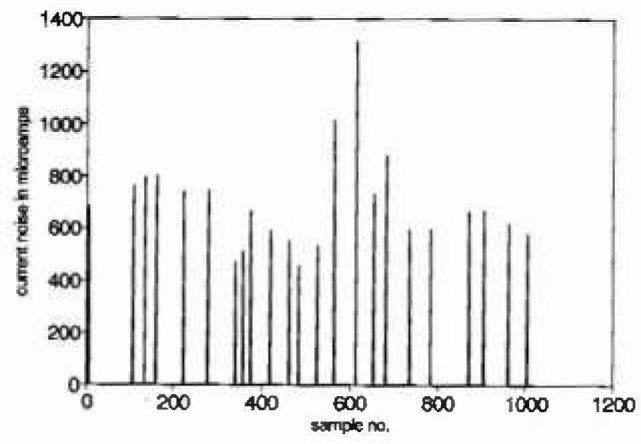
Isolated peaks in current noise measurements.

**Fig. 1(c):**
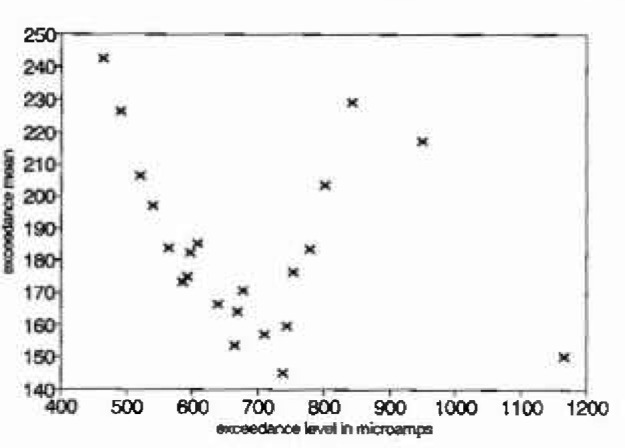
Mean excess plot for current noise measurements.

**Fig. 2(a):**
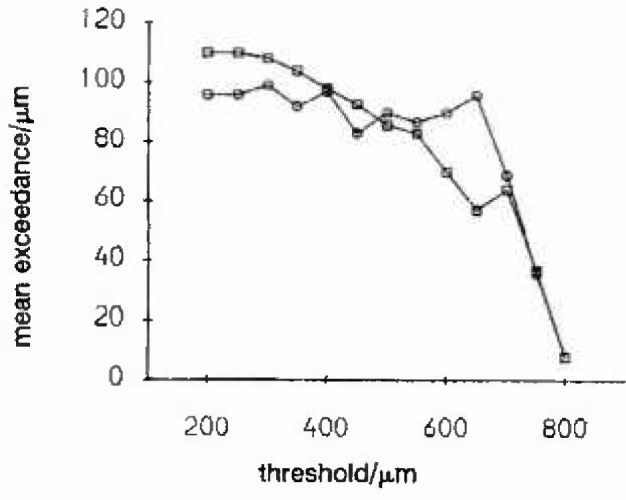
Mean excess plot for typical 26 week basalt groundwater coupon profile: ○–mean declustercd exceedances; □–mean of all exceedances.

**Fig. 2(b):**
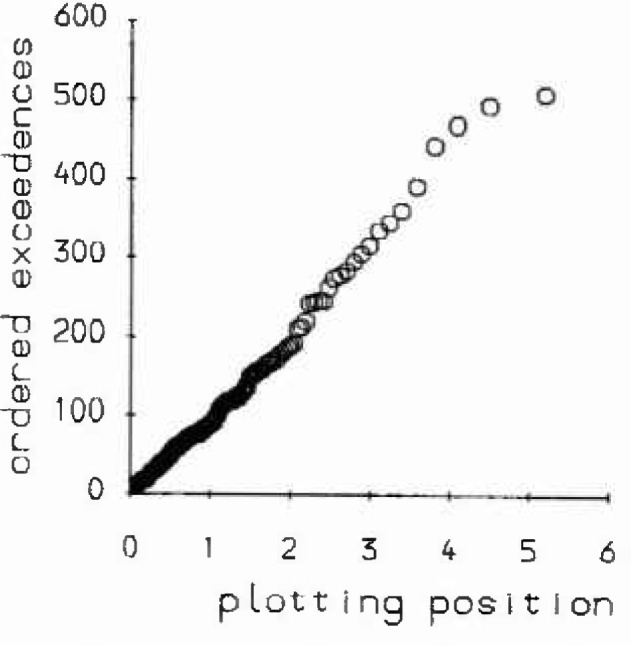
Exponential probability plot of declustered exceedances above 300 μm.

**Table 1:** Pit depths above 6 μm in stainless steel sheet college roofs (area 500 m^2^; samples 10 cm^2^; thickness 400 μm)

Roof 1 (50 months)
131 106 35 26 26 25 23 20 20 18 18 18 17 16 16 15 15 15 14 14 14 14 14 14 14 14 14 12 12 12 12 12 10 10 8 8 8 8 8 8 8 8
Roof 2 (29 months)
140 106 95 77 72 55 55 53 52 36 33 32 32 30 28 28 26 26 25 24 24 24 22 22 20 18 18 16 16 16 16 14 14 12 12 12 8 8 8

**Table 2:** Summary of model fitting and prediction using maximum likelihood for the generalized Pareto distribution for a typical 26 week basalt groundwater coupon profile

Threshold	Mean cluster excccdance (μm)	Number of clusters	λ	*σ*	*ξ*	*q*_25_	*q*_250_
300	99	177	4425 (333)	98.0 (11)	0.01 (0.08)	1158 (260)	1406 (430)
350	92	146	3650 (302)	99,0 (11)	0.04 (0.09)	1205 (300)	1500 (527)
400	97	96	2400 (245)	104.3 (16)	−0.08 (0.11)	1004 (214)	1102 (322)
450	83	76	1900 (218)	83.4 (11)	−0.01 (0.13)	1057 (241)	1233 (405)
500	90	50	1250 (177)	102.6 (23)	−0.14 (0.17)	963 (213)	1037 (309)
550	87	29	725 (135)	108.5 (12)	−0.23 (0.31)	918 (250)	961 (339)
